# Supplementary material for: Giant valley splitting in monolayer WS2 by magnetic proximity effect
Source: Nat Commun. 2019 Sep 13;10:4163. doi: 10.1038/s41467-019-11966-4 (PMC6744439; doi:10.1038/s41467-019-11966-4)
Supplement: Supplementary file 1 — Supplementary Information [file 41467_2019_11966_MOESM1_ESM.pdf]

# Supplementary Information

# Giant Valley Splitting in Monolayer $WS_2$ by Magnetic Proximity Effect

Tenzin Norden[1], Chuan Zhao[1], Peiyao Zhang, Renat Sabirianov[2],

Athos Petrou[1], and Hao Zeng[1]

[1]Department of Physics, University at Buffalo, the State University of New York, Buffalo, NY 14260, USA;

[2]Department of Physics, University of Nebraska-Omaha, Omaha, NE 68182, USA

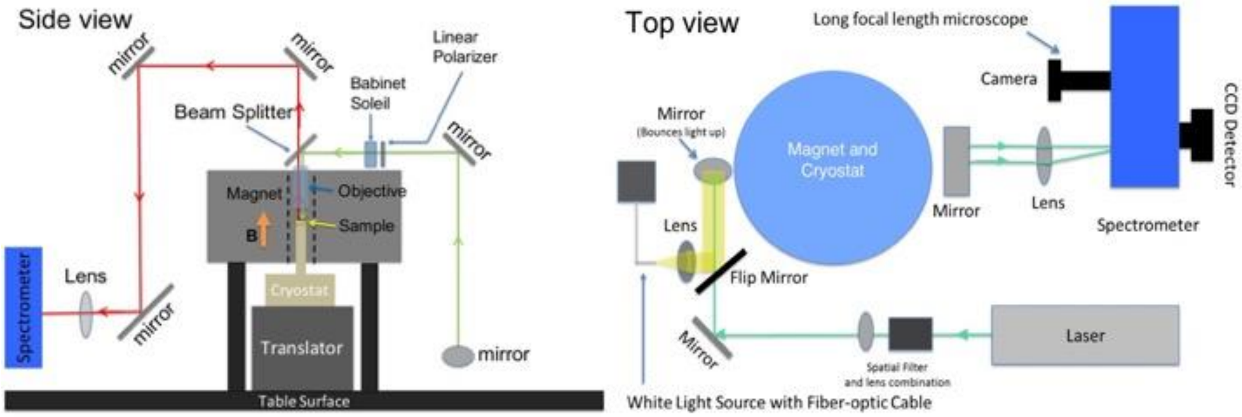


**Supplementary Figure 1. Magneto-reflectance measurements.** A schematic of the setup for magneto-reflectance measurements. Positive magnetic field is defined as the upward direction. The incident light is right and left circularly polarized by a combination of a babinet soleil and a linear polarizer. The reflected light from the sample is collected using a microscope objective and the light was then focused onto the entrance slit of a single monochromator that uses a cooled charge-coupled device detector array.

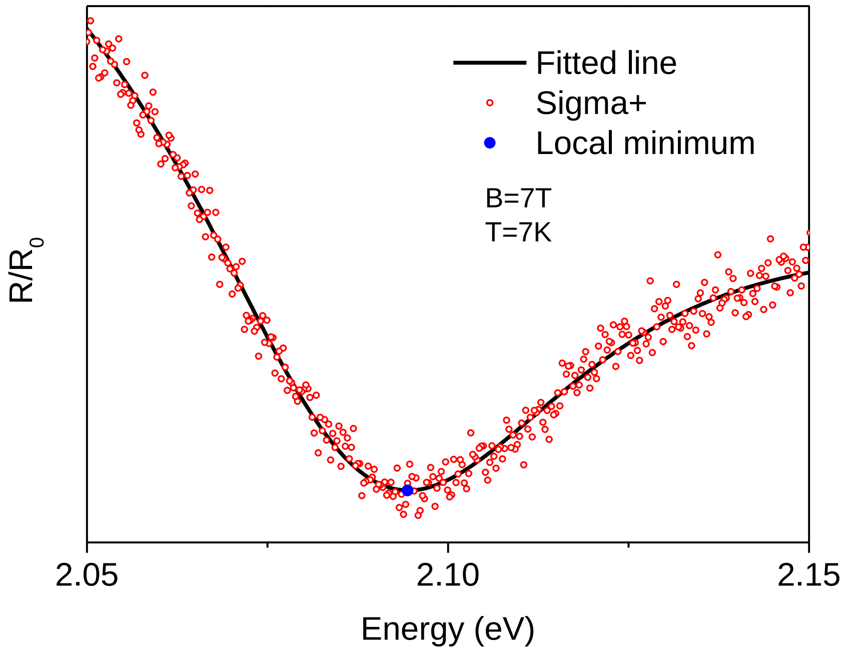


**Supplementary Figure 2. Magneto-reflectance curve fitting.** A reflectance spectrum of monolayer $WS_2$ with sigma+ incident light. The red dots represent the data points and the black line represents the fitted curve. The blue dot is the fitted peak position.

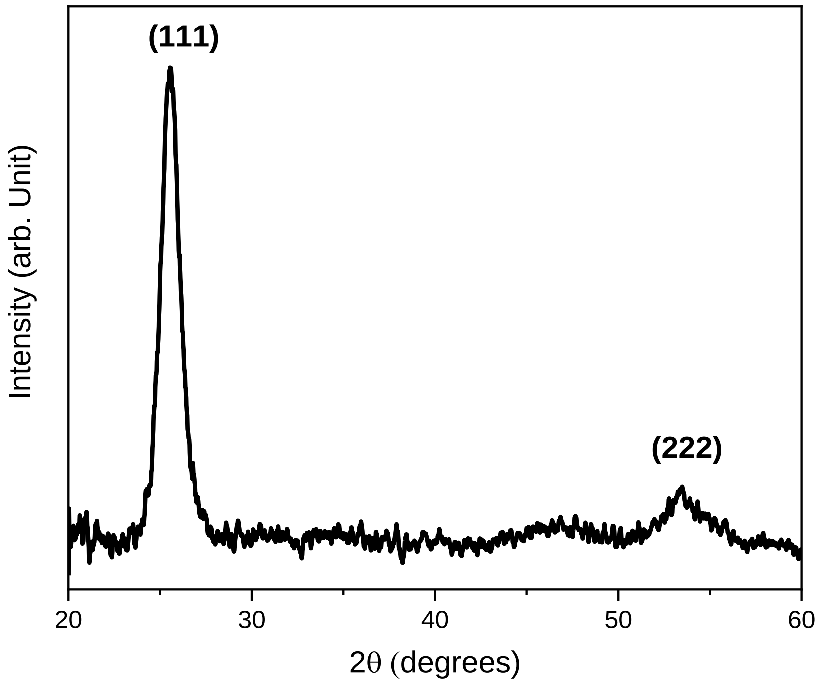


**Supplementary Figure 3. X-ray diffraction (XRD) pattern of 10 nm thick EuS film on Si.** The XRD pattern of a 10 nm thick EuS layer grown on Si substrate. Only (111) and (222) peaks are clearly visible, suggesting (111) orientation of the film.

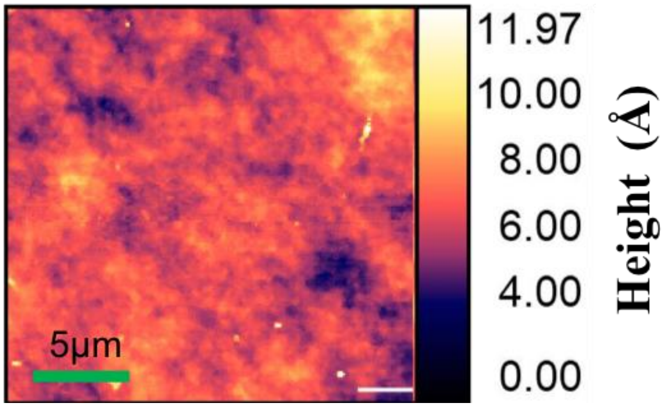


**Supplementary Figure 4. Atomic force microscope (AFM) image of the EuS surface.** An AFM image of a 10 nm thick EuS film on Si substrate. The root mean square roughness of the EuS surface is 0.92 nm.

## Supplementary Note 1: Magneto-reflectance curve fitting

For reflectance signal, it is conventional to use absorptive and dispersive line shape in addition to a linear function to fit the curve and use the local minimum as the peak position as shown in supplementary Fig. 2.

The fitting equation is:

$$f(x) = A\frac{(q\frac{m}{2} + x - \mu)^2}{(\frac{m}{2})^2 + (x - \mu)^2} + kx + b,$$

here $A$ is the amplitude, $q$ is the Fano parameter which represents the ratio of resonant scattering to the background scattering, $m$ is the width of the line shape, $k$ is the linear slope and $b$ is the intercept.
